# Autogenously Calcined Clays from Coal Tailings Dumps as Supplementary Cementitious Material: From Exploratory Investigations to an Industrial Trial

**DOI:** 10.3390/ma18050993

**Published:** 2025-02-24

**Authors:** Steffen Overmann, Anya Vollpracht

**Affiliations:** Institute of Building Materials Research (ibac), RWTH Aachen University, Schinkelstr. 3, 52062 Aachen, Germany; vollpracht@ibac.rwth-aachen.de

**Keywords:** calcined clays, tailings dump, coal mining, pozzolan, supplementary cementitious material, blended cement

## Abstract

Autogenously calcined clays from tailings dumps, which are formed by the ignition of the contained residual coal, represent a huge resource of potentially pozzolanic material worldwide. This work presents preliminary studies of samples from the Western coal regions in Germany and investigations on the first industrially produced cement with autogenously calcined tailings concerning its suitability as supplementary cementitious material (SCM). Samples of the tailings materials and blended cements were thoroughly characterized physically, chemically and mineralogically. The reactivity was studied using the R^3^ test and mortar compressive strength testing. The influence on cement hydration was studied using X-ray diffractometry (XRD) and isothermal calorimetry. The preliminary investigations showed that the material is basically suitable as SCM, as it consists mainly of SiO_2_ and Al_2_O_3_, which partially occurs in X-ray amorphous form and has a low content of impurities, which can impair cement properties such as carbon or sulfur. The R^3^ reactivity and the relative compressive strength differed significantly depending on the sample. For the industrial cement production trial, low-processed material was used which still contained inert fractions. The average sample showed a relatively low R^3^ reactivity but still significantly higher than mostly inert materials such as limestone or quartz powder. Calorimetry and XRD investigations on cement pastes showed that the clinker reactions remain basically unaffected by the tailings. Mortar tests showed that the material contributes to strength development at a late age. It can be concluded that the tailings are basically suitable as SCM and, in appropriate blends, the clinker factor and, thus, the CO_2_ footprint of cement can be reduced.

## 1. Introduction

This study deals with the use of tailings from coal mining, which are landfilled in coal mining areas worldwide. Due to possible pozzolanic properties resulting from the often-occurring dump fires, the tailings material can be used as a supplementary cementitious material (SCM). The following introduction describes the origin of the tailings, their possible impact on the environment, the cause of dump fires and the potential of burnt tailings as SCM for the building materials industry.

Coal tailings result from the mine workings, coal extraction and coal preparation. The tailings portion of the raw mining coal is not a constant quantity. Initially, the coal was hewn by hand from the bedrock, and the bedrock was left standing. Thus, the proportion of waste rock was very low compared to coal production. As a result of progressing mechanization, the proportion of waste rock has grown steadily over the years of mining since coal was no longer mined selectively but together with the waste rock [1].

Hard coal was formed over millions of years from dead plant remains by the deposition of sedimentary layers and the associated increase in pressure and temperature. As a result, the tailings that arose during coal processing consist mainly of shales, clay, sandstones, limestone, gypsum, remaining coal and sulfur compounds [2]. The proportion of coal in the tailings varies depending on the mining and underground construction method selected. In the construction phase of the mining operation large coal contents remained in the waste rock delivered to the tailings dumps. Furthermore, the coal content in the tailings varied according to the processing method selected. As in mining, mechanization also progressed in processing. In the early days of coal mining, only hand-picking (manual separation) was carried out. In this process, coal particles below a grain size of approx. 20–30 mm remained in the tailings. With progressive mechanization and the use of setting machines, float-sink sorting and flotation, the coal output has been increased, and the amount of coal remaining in the tailings has been steadily reduced. According to the current state of the art and choice of process, the yield of hard coal extraction is between 85–95%. This means that even when all grain classes are included in the processing, considerable amounts of coal still remain in the tailings.

**Figure 1:**
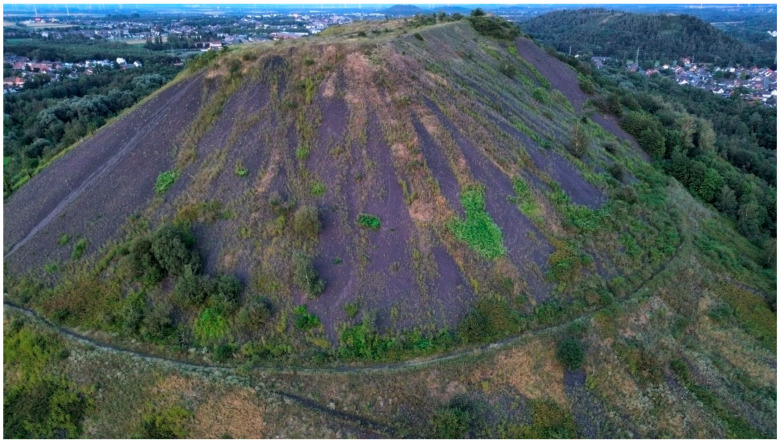
Tailings dump Noppenberg, Herzogenrath, Germany.

In Germany, there are seven coalfields covering large parts of North Rhine-Westphalia (Ruhr area, Aachen, Ibbenbühren) as well as Saarland and Saxony (Zwickau, Lugau-Oelsnitz, Döhlen coalfields). The German federal ministry for the environment, nature conservation and nuclear safety (BMU) estimates the mass of tailings in the area of today’s Germany that were stockpiled between 1792 and 2003 to be about 3.6 billion tons [1]. For a potential use of the tailings as secondary raw material in the construction industry, this thus represents a considerable resource. Figure 1 shows an example of a tailings dump.

Although some tailings dumps are considered national heritage sites as well as recreational areas or nature reserves, the tailings dumps also represent a significant interference with the original natural landscape, which can have a negative impact on the environment and require costly permanent monitoring. The tailings usually have good water permeability, which means that the tailings body and its surface are very dry, whereas water accumulates at the bottom of the tailings. Man-made ponds are used to collect pollutant-enriched leachate. Physical weathering leads to increased dust emission from the tailings dumps as well as erosion by water, wind and gravity-induced processes. The leachate from the tailings dumps affects water bodies and vegetation in the vicinity of the dump depending on its composition. Weathering leads to chemical changes that affect the constituents of the leachate. In the initial weathering phase, generally, salts and sulfates are dissolved, increasing the sulfate and chloride content in the leachate. In the course of further weathering, oxidation of the pyrite, which has been enriched by processing, occurs, resulting in the formation of sulfuric acid, which dissolves other minerals (e.g., clays, carbonates) and mobilizes sulfates, aluminum and heavy metals. The pH of the leachate is considerably reduced, and, as a further consequence of pyrite weathering, an anthropogenically acidified soil is formed [1]. In addition, the stability of the tailings dump must be ensured, which is why, in many cases, remediation of the tailings dump bodies is carried out. All these factors, in combination with the following described potential of the tailings as secondary raw material for the building material industry, speak for an at least selected retreat of tailings dumps.

Many of the tailings have been exposed to increased temperatures due to spontaneous combustion of the coal residues or are currently burning, which is a worldwide phenomenon. The exothermic process of oxidation of the coal residues contained in the tailings, often in interaction with the oxidation of pyrite as a typical accompanying mineral, leads to spontaneous combustion of the tailings under suitable boundary conditions (oxygen supply, temperature, pressure, coal content) [3,4]. Furthermore, human interference with the heap body is often the cause of fires. The influence of temperature on the originally clayey tailings potentially leads to pozzolanic properties and thus provides the basis for the use as supplementary cementitious material (SCM), e.g., the BMU states the average proportion of clay minerals in the tailings dumps of the German Ruhr area to be 59–73 wt.% [1].

Currently, an international research focus on alternative SCM is on clayey sediments and sedimentary rocks [5,6], which are abundant worldwide. The clay minerals can develop pozzolanic properties by thermal treatment (calcination) between ~600 and 950 °C (depending on the composition), i.e., Si and Al can dissolve in the alkaline environment of the cement paste and form C-(A-)S-H phases with the calcium and water present, similar to the strength-building phases in a pure Portland cement system. To date, research on calcined clays has focused on the potential of raw clays and the suitable utilization of these as SCM by means of technically controlled calcination. When calcined clays are used to substitute a part of the clinker, the chemically induced CO_2_ release that occurs in Portland cement production is reduced, and CO_2_ and energy are also saved by reduced firing temperatures compared to clinker production.

With the use of uncontrolled, “autogenously” calcined materials obtained from tailings dumps, which originally contained clay mineral phases as major constituents, additionally, the energy and resource consuming process of controlled calcination can be avoided. However, the factors influencing clay reactivity in a cementitious system are very complex and the properties of calcined mixed clays resulting from their varying raw phase compositions and their temperature behaviors are still not understood in detail [7].

The most frequently occurring clay minerals or clay mineral groups are kaolinite, smectite (mainly montmorillonite) and illite. After calcination at a suitable temperature, kaolinite shows the highest reactivity, followed by montmorillonite. Calcined illite shows the lowest reactivity potential but is still sufficient to be used as an additive. The optimum calcination temperature for kaolinite is between 600 and 900 °C, with the resulting reactivities hardly differing in this range [8,9,10]. Above about 600 °C, dehydroxilation leads to lattice stresses and results in an X-ray amorphous structure, while the grain morphology is largely preserved. From this metastable structure, aluminum and silicon can easily be dissolved in the cementitious environment, and strength-building C-A-S-H phases can be formed. From about 925 °C, first recrystallizations occur in the form of Al spinel, reducing the reactivity. With further temperature increase, mullite is formed [11]. The montmorillonite structure, in contrast, remains stable after dehydroxilation. Amorphization and thus the occurrence of a metastable, reactive phase takes place only at temperatures between 800 and 900 °C, depending on the composition, through sintering or melting processes. The temperature window with high reactivity is very small since recrystallizations also occur again with the onset of melting [10,12]. Illites behave similarly when exposed to temperature. Dehydroxilation does not lead to amorphization, but at temperatures between approx. 900 and 950 °C, a reactive amorphous phase is formed by sintering or melting processes, from which minerals recrystallize again with a flowing transition. Reactivity is strongly reduced at temperatures above 950 °C. Furthermore, it is stated in the literature that CaO present, which is formed, for example, during the co-calcination of calcite or dolomite, reduces the melting and recrystallization temperature [13,14,15]. The different processes occurring during calcination for the different main clay minerals are summarized in a simplified manner in Table 1 according to [7].

The reactivity of the amorphous phases depends on their chemical composition and structure. The reduction in reactivity due to the crystallization of Al-/Si-rich mineral phases at high temperatures is based on the assumption that the Al or Si ions, which were previously easily soluble in the metastable phase, are incorporated into a thermodynamically more stable crystal lattice. However, there are no detailed studies on how inert/stable these recrystallized phases are in cementitious systems or how the properties of the residual amorphous structure are affected. In addition, depending on the chemical composition, a reactive melt phase may again be formed when the temperature is further increased, e.g., for pure mullite, this temperature is ~1800 °C [16]. However, other mineral components, such as feldspars, can act as fluxes and reduce the melting temperatures [17].

Since the original material composition and the temperature exposure of uncontrolled calcined clays from tailings dumps are unknown, it is even more difficult to estimate their potential on the basis of characteristic values that can be determined quickly. The temperature in tailings dumps ranges up to ~1500 °C according to reference [18], depending on the original distribution of the coal residues, the oxygen supply, and the position of the fire in the dump body. Also, the duration of temperature influence cannot be spatially resolved. The dump fire itself often lasts for decades. In addition, the initial mineralogy may have differed significantly on a small scale since the tailings consist of fragments of waste rock piled together.

Much of the literature concerns phase formations in the surrounding rock during the burning of coal seams, e.g., [3,19,20,21], which is approximately comparable to the burning of tailings. There are also particular studies on burned tailings with mineralogical investigations [22,23] and on the use of tailings as SCM after artificial calcination [24]. However, studies on the use of autogenously calcined tailings as SCM are rare. In [18], the reactivity of autogenously calcined tailings as a binder component was demonstrated by a significant compressive strength contribution in concrete. Overall, the findings of the literature on the composition of autogenously calcined sediments associated with coal fires are very diverse, as the boundary conditions of phase formations are highly variable (initial composition, temperature, pressure, atmosphere). Reliable, in-depth studies on the suitability of a reactive binder component do not exist.

Therefore, this study summarizes the pioneering work from exploratory investigations on the suitability of selective samples from different tailings dumps to a pilot trial of cement production on an industrial scale. In the first section, the ranges of different sample characteristics, such as chemical and mineralogical composition, as well as relative compressive strength and R^3^ reactivity [25], are discussed. Based on the positive preliminary results an industrial trial was realized. Material was mined, processed and blended cements were produced in an industrial plant. The second section shows the investigations into the tailings material for the industrial trial and the produced cement. Additionally, model mixtures also with further decreased clinker factors were investigated to thoroughly evaluate the suitability of the material for use as a cement component.

## 2. Materials and Methods

### 2.1. Materials

#### 2.1.1. Exploratory Investigations in Section 3.1

For the preliminary investigations in Section 3.1, samples were selectively taken from four different locations in western Germany. These were either hand-picked from the surface of the dump, taken by surface pitting or via drill core extraction. They were investigated as average samples, hand-picked fractions or processed according to patent WO 2021/224094 [26]. For reactivity and relative strength testing, the samples were ground using a vibratory disk mill or laboratory ball mill. In most cases, to compare the fineness, only the Blaine values were determined, which were in the range of 5000 to 10,000 cm^2^/g for all samples. It should be noted that the Blaine value was only used as an indicator of fineness, as values > 6000 cm^2^/g and the occurring layer silicates can significantly increase the measurement error. The samples were examined for exploration and not systematically analyzed; thus, not all investigations were performed on the different samples (such as chemical and mineralogical analysis, mortar compressive strength testing and R^3^ reactivity, as described in Section 2.1.2 and Section 2.2). Therefore, only the ranges of the results are presented in the results and discussion section (see Section 3.1)

#### 2.1.2. Industrial Trial in Section 3.2

The tailings sample for the industrial cement production trial in Section 3.2 was provided from a tailings dump in Saarland, Germany, where, due to restructuring work, an operation was carried out in the heap body, and the material could be removed without applying for complex mining permissions. The sample for the investigations on single fractions in Section 3.2.1 was taken in several sub-samples (~60 kg in total). They were taken from the running conveyor at the output of a processing operation of about 1000 tons from a side part of the heap body to obtain a sample that is as representative as possible. However, since the properties of individual grains are investigated, as described in the following, the representativeness of the sample is not relevant. For project-specific reasons, only a low degree of processing was applied. Approx. 100 tons of the processed material were provided in an air-dried condition (water content ~3.5 wt.%) for the industrial cement production trial in Section 3.2.2.

##### Selective Fractions

Since the tailings material for the industrial trial consisted of very heterogeneous grains, visually well-distinguishable grains were handpicked and categorized. Of each defined category, about 50–150 g were selected. The characteristics of each category are shown in the discussion Section 3.2.1. The 60 kg sample was not completely divided into individual fractions since most of the grains exhibited characteristics of several different defined fractions. After photo-taking, optical description and determining the skeletal density as described in Section 3.2.1, the fractions were crushed for further analysis using a jaw crusher and then ground to a grain size of <125 µm using a vibratory disk mill made of tungsten carbide. The grinding program was 30 s grinding—5 min cool down—30 s grinding—sieving the fraction < 125 µm and repeating the program with the fraction >125 µm until the whole sample passes the 125 µm sieve to minimize thermal–mechanical influences on the sample.

##### Industrial Cement Production Trial

During the cement production, the clinker was ground in the industrial ball mill together with the sulfate carrier, limestone (target value of 5 wt.%), a grinding aid, and the tailings material. According to the cement standard EN 197-1 [50], pozzolans can be assigned as “P: natural pozzolan” or “Q: natural tempered pozzolan”. Although the autogenously calcined tailings material was not actively tempered, it is referred to as Q in the following. Q was fed in four different stages in which the proportion was progressively increased (10, 15, 20, 25 wt.%). The grinding process was adjusted each time to achieve a comparable particle size distribution, which was measured continuously during grinding. Approx. 150 tons of cement were produced with 20 wt.% of Q for further application. The cements with 10, 15 and 25 wt.% were only produced for the laboratory tests, and after sampling, the remaining cements were diverted to a separate silo. Representative samples of about 30 kg of each produced cement were taken from the running conveyor for the presented investigations.

##### Model Mixtures

To analyze the influence of Q in the cement system more precisely, model mixtures were investigated. A CEM I 42.5 R was used as base cement. A representative sample of Q for the investigations of model mixtures was ground with a laboratory ball mill. The ground calcium carbonate (GCC) was a ground marble and was used to compare the performance of Q with the quasi-inert GCC. For investigations with lower clinker factor, ternary systems with granulated blast furnace slag (GGBS) were designed. The GGBS was taken from the cement plant and ground in a laboratory ball mill. Table 2 gives an overview of the samples used in the different chapters.

Table 3, Table 4, Table 5, Table 6, Table 7 and Table 8 show the characterization of the materials of the industrial trial and of the model mixtures. For analysis, the samples, except cements and clinker, were dried at 105 ± 5 °C to constant mass. For the mineralogical characterization, subsamples were further ground by hand using an agate mortar to a grain size < 63 µm. The mineralogical composition was determined using an X-ray diffractometer Panalytical X’Pert Pro with an X’Celerator detector (co. Malvern Panalytical GmbH, Kassel, Germany). Data were acquired in a range of 5 to 70° 2θ with a step size of 0.0167° 2θ and a total measurement time of 2 h. Rietveld refinement was performed to determine the quantitative composition of the samples using the software Panalytical Highscore Plus 4.8. For the quantification of the amorphous content, the internal standard method was applied using a rutile standard. 

The loss on ignition was determined according to EN 196-2 [27] on samples of 1.00 ± 0.05 g at 950 °C in a muffle furnace. To analyze the carbon and sulfate contents, a carbon/sulfur analyzer, Eltra CS-2000 (co. ELTRA GmbH, Haan, Germany), was used. For this, 0.150 ± 0.001 g of the sample was weighed in an annealed ceramic crucible and covered with 0.7 g iron granules and 2.0 g tungsten granules. The crucible was sealed and put into the induction furnace for subsequent combustion at temperatures above 1800 °C in a pure oxygen stream. A double determination was performed. Further chemical analysis was performed by X-ray fluorescence using a Panalytical Axios X-ray fluorescence spectrometer (co. Malvern Panalytical GmbH, Kassel, Germany): 0.8 g of annealed samples of the samples mixed with 8.0 g lithium tetraborate LT100 (co. XRF Scientific Europe GmbH, Karlstein, Germany) to produce fused tablets using a Vulcan fusion machine 4MA (co. PD Instruments, Toszek, Poland).

**Table 5:** Chemical composition of the raw materials and cements for the industrial trial.

Phase	Unit	Q	Sulfate Carrier	Limestone	Clinker	CEM II/A-Q		CEM II/B-Q	
						Q = 10%	Q = 15%	Q = 20%	Q = 25%
LOI	wt.%	3.54	6.95	36.68	0.48	2.61	3.39	3.52	2.98
total sulfur as SO_3_		0.70	41.72	0.57	1.45	3.56	3.49	3.37	3.42
total carbon as CO_2_		4.24	4.87	37.01	0.20	2.45	3.47	3.44	2.87
chloride		-	-	-	-	0.02	0.03	0.03	0.02
unsoluble, HCl/KOH		-	-	-	-	8.93	12.52	16.69	20.03
Na_2_O		0.18	1.43	0.11	0.17	0.18	0.17	0.28	0.17
K_2_O,		4.21	1.00	0.79	1.30	1.64	1.76	1.84	2.01
Na_2_O equivalent		2.95	2.09	0.63	1.03	1.25	1.33	1.49	1.50
MgO		1.62	2.56	1.17	1.82	1.72	1.71	1.60	1.74
Al_2_O_3_		23.92	3.35	3.64	6.01	7.22	7.87	8.49	9.28
SiO_2_		57.78	11.44	10.00	20.10	22.69	23.96	24.60	27.84
P_2_O_5_		0.07	0.04	0.03	0.19	0.16	0.15	0.15	0.15
CaO		0.87	33.50	44.83	64.29	55.51	53.04	50.36	47.54
TiO_2_		1.03	0.16	0.13	0.28	0.34	0.38	0.38	0.45
MnO		0.12	0.03	0.03	0.07	0.07	0.08	0.06	0.09
Fe_2_O_3_		6.20	1.31	1.30	3.90	3.84	3.92	3.98	4.12

**Table 6:** Chemical composition of the additional raw materials for the model mixtures.

Phase	Unit	CEM I 42.5 R	GCC	GGBS
LOI	wt.%	2.25	-	−0.52
total sulfur as SO_3_		3.26		2.80
total carbon as CO_2_		1.87	43.5	1.22
chloride		0.02	-	0.01
unsoluble, HCl/KOH		1.07		0.15
Na_2_O		0.2	0.07	0.30
K_2_O		0.78	0.07	0.73
Na_2_O equivalent		0.71	-	0.78
MgO		1.55	1.50	7.40
Al_2_O_3_		5.55	0.21	11.64
SiO_2_		21.21	0.95	36.64
P_2_O_5_		0.11	-	0.02
CaO		62.75	53.6	41.38
TiO_2_		0.31	-	0.68
MnO		0.07		0.50
Fe_2_O_3_		2.43	0.10	0.75

The particle size distribution was determined by a laser granulometer Bettersizer S3 Plus (co. 3P Instruments GmbH & Co. KG, Odelzhausen, Germany). The true density was determined with the helium pycnometer AccuPyc 1330 (co. Micromeritics GmbH, Unterschleißheim, Germany), and the specific surface area was determined via the 5-point BET method with a 3P sync 210 (co. 3P Instruments GmbH & Co. KG, Odelzhausen, Germany) using N_2_ as measuring gas. The Blaine fineness was determined according to EN 196-6 [28].

**Table 7:** Physical characterization of the industrial trial cements.

Cement	True Density	Blaine Value	BET Surface	Grain Size Percentiles		
				d_10_	d_50_	d_90_
	g/cm^3^	cm^2^/g		µm	µm	µm
CEM II/A-Q10	3.12	3890	14,300	2.2	16.6	59.1
CEM II/A-Q15	3.08	4030	14,600	2.2	16.4	58.6
CEM II/B-Q20	3.04	4160	18,100	2.2	15.9	57.0
CEM II/B-Q25	3.04	4130	17,300	2.2	15.8	56.5

**Table 8:** Physical characterization of the raw materials for the model mixtures.

Material	True Density	Blaine Value	BET Surface	Grain Size Percentiles		
				d_10_	d_50_	d_90_
	g/cm^3^	cm^2^/g		µm	µm	µm
CEM I 42.5 R	3.14	3200	10,870	0.4	9.4	36.8
Q	2.69	5990	35,485	1.1	8.6	40.5
GCC	2.73	13,500	35,010	0.4	2.1	6.6
GGBS	2.93	5050	14,625	0.5	9.2	36.9

### 2.2. Methods

#### 2.2.1. Scanning Electron Microscopy

Scanning electron microscopy (SEM) was used to investigate tailings samples in ground form. It was only applied to samples from the exploratory investigations in Section 3.1. Loose powders were examined as well as polished sections of the powders embedded in epoxy resin. The samples were coated with carbon before being introduced to the sample chamber of the SEM (Gemini SEM 300, co. Carl Zeiss AG, Oberkochen, Germany). For visual analysis, a secondary electron (SE) and a backscattered electron detector (BSE) were used. Chemical analysis was performed using energy-dispersive X-ray spectroscopy (EDX) integrated in the SEM.

#### 2.2.2. Determination of Grain Skeletal Density

The grain skeletal density was determined for the ten fractions of Q used in Section 3.2.1. They were first washed under flowing tap water to remove dirt residues and were then dried at 60 °C to mass constancy. The skeletal density was determined using buoyancy weighing according to the Archimedean principle. Hereby, at first, the dried grain fraction was weighted. Then, a device was placed on the balance, holding a basket that dives in a tap-water-filled vessel via a lateral arm. The sample was placed in the basket underwater so that the weight of the sample underwater could be determined. The difference between the mass of the sample in air and under water is similar to the mass of water displaced. To calculate the sample volume, the density of the tap water at 20 °C was assumed to be 1 g/cm^3^. The grain skeletal density was determined with the grains saturated for 24 h. It should be emphasized that in difference to the bulk density, open pores are not included in this measuring approach for skeletal density; however, with regard to practical preparation processes, open pores are also water-filled during the wet processing of density sorting.

#### 2.2.3. Isothermal Calorimetry

The reactivity of the samples of tailings material and, for comparison, of GCC in Section 3.2.1 and Section 3.2.3 was determined according to ASTM C1897-20 [25] using isothermal heat flow calorimetry (TAM Air, co. TA instruments, New Castle, DE, USA). A potassium solution was prepared by mixing 4.00 g of potassium hydroxide and 20.0 g of potassium sulfate in 1.00 L of deionized water. Moreover, 5.00 g of the sample was premixed with 15.00 g Ca(OH)_2_ and 2.5 g of CaCO_3_ by shaking in a closed vessel. After tempering all components at 40 °C, 27.00 g of the potassium solution was added to the solids and mixed with a shear blender at 1600 rpm for 2 min. Subsequently, each 15 g was weighted in two sample vessels for double determination. The samples were immediately placed in the calorimeter, and the reaction heat was recorded for 7 days. According to the ASTM standard, the first 75 min were neglected in the evaluation.

The reaction heat of the four industrially produced CEM II cements was also investigated using isothermal heat flow calorimetry (TAM Air, TA instruments). The tests were performed at 20 °C with a w/c ratio of 0.5. After adding water to the cements, they were mixed with a shear blender at 1600 rpm for 2 min. Subsequently, each 15 g was weighted in two sample vessels for double determination.

#### 2.2.4. Hydration Study Using XRD

CEM II/A-Q10 and CEM II/B-Q20 as well as the model mixture using the CEM I 42.5 R with 20 wt.% substitution by Q and the pure CEM I 42.5 R as reference were investigated in a hydration study on cement paste with a w/c ratio of 0.5, adapting the method described in [29]. The solids were weighed in and premixed at 600 rpm with a shear blender for 2 min. Then, the water was added, and the cementitious paste was mixed for further 3 min at 1600 rpm. A cylindric silicon mold with an inner diameter of 25 mm and 100 mm height was filled with the paste. To compact and de-aerate the paste, the formwork was carefully tapped on the table. Care was taken to avoid segregation in order to keep the effective w/b ratio constant over the height. The mold was then closed with tape and cured in an overhead rotator at a rotation speed of 5 rpm to further avoid segregation. After 24 h the sample was removed from the mold. The top of the sample was cut-off, crushed and placed in the sample container. Deionized water was added for the storage of the rest of the sample. The crushed slice helps to minimize the leaching while keeping the sample water-saturated. For the storage, the sample container was closed tightly by wrapping Parafilm with tape to prevent carbonation. At the measuring dates (age of 1, 2, 7, 28 and 90 days), two slices of about 2 mm were cut from the top of the cylindric sample using a precision saw. The first slice was discarded. The other was briefly rinsed with tap water to remove residues from sawing and directly processed for the XRD measurement. The slice was polished immediately before measurement using a few drops of deionized water on a silicium carbide abrasive paper to obtain a fresh surface. The surface was then briefly rinsed with deionized water and directly surface dried with a paper sheet. The mineralogical analysis was performed with an X-ray diffractometer Panalytical X’Pert Pro with an X’Celerator detector (co. Malvern Panalytical GmbH, Kassel, Germany). The data were recorded in a range from 6 to 55 °2θ with a step size of 0.0334 °2θ with a total measuring time of 30 min. For the determination of the X-ray amorphous content an external rutile standard was used. The quantitative phase composition was calculated by Rietveld refinement software Highscore Plus 4.8 (co. Malvern Panalytical GmbH, Kassel, Germany). It should be noted that although rutile is a typical standard in cement analysis, its mass absorption coefficient is significantly higher than that of cementitious pastes and increases the inaccuracy of the results [29].

#### 2.2.5. Setting Time, Soundness and Mortar Experiments

The water demand and setting time were determined according to EN 196-3 [30] using the Vicat needle device. The soundness was determined according to EN 196-3 [30] using the Le Chatelier ring. Mortars were prepared according to EN 196-1 [31] using the four produced CEM II cements with water, cement and standard sand in a ratio of 0.5:1:3. The compressive strength was determined at 2, 7, 28 and 90 days. The mortar flow of separately prepared mortars was investigated directly after mixing as well as after 10, 30, 60 and 90 min using a Hägermann cone by applying 15 shocks according to EN 1015-3 [32].

Further mortars were prepared similarly for compressive strength testing using model mixtures in Section 3.2.3; however, the binder compositions were varied as clearly indicated in the results chapter by a combination of CEM I 42.5 R with the tailings material (Q) or ground calcium carbonate (GCC) or a combination of the CEM II/B-Q with ground granulated blast furnace slag (GGBS).

## 3. Results and Discussion

### 3.1. Exploratory Investigations on Selective Samples from Different Dumps

**Figure 2:**
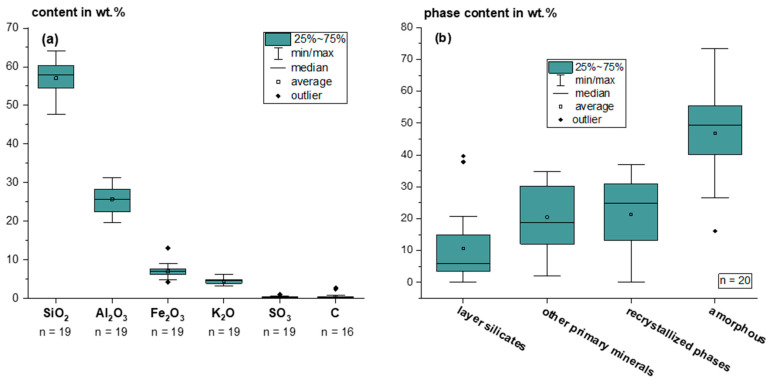
Ranges of chemical composition (**a**) and mineral phase composition (**b**) of the tailings samples.

As part of the exploration of the potential of autogenously calcined tailings material as SCM, samples were selectively taken from various tailings dumps in western Germany, as described in Section 2.1. Figure 2 shows the ranges of composition.

Chemically, the tailings materials mainly consist of Si, Al and Fe, which is basically suitable for use as SCM. Low carbon and sulphate contents indicate that the dump is sufficiently calcined, and these substances will not cause any problems in the cement system. Mineralogically, the burnt tailings consist of primary minerals, such as uncalcined layer silicates (here muscovite, illite and kaolinite) and others (predominantly quartz, feldspar and hematite), probably recrystallized minerals (predominantly mullite, cordierite and spinel), and an amorphous phase which content usually ranges between 40 and 60 wt.% and indicates a potential for pozzolanic reactivity. The distinction between primary minerals (considered here as minerals in the dump prior to heat exposure) and recrystallized minerals, which form from the primary minerals at high temperature, is not always definite since the source material as sedimentary rock can contain various minerals as residues of rock weathering. In addition, typical primary minerals such as quartz and feldspar can also form through recrystallization when temperature decreases from high values, as they usually crystallize from felsic magma [33]. Hematite can be caused by sedimentary processes but can also be formed from iron-rich typical sedimentary iron hydroxides such as goethite or iron sulfides such as pyrite or marcasite at high temperatures [34,35,36,37]. The absence of these minerals, in combination with the occurrence of hematite, suggests that the tailings were exposed to elevated temperatures. However, the hematite formation temperature is relatively low compared to the amorphization and recrystallization temperature of other minerals. However, certain quantities of typical high-temperature minerals such as mullite and cordierite, which occur together with the amorphous phase, clearly indicate exposure to more than 900 °C of temperature [11,38,39]. Since the thermodynamics of mineral transformation are very complex and depend on the exact chemical and mineralogical composition and the parameters of heat exposure, the defined mineral phase inventory cannot yet be assigned to specific temperature conditions. With regard to reactivity, the properties of the amorphous phase are decisive, as further discussed in Section 3.2.1. It is, therefore, expedient to enrich the amorphous portion by processing the raw tailings. In the first trials, it was possible to higher the amorphous content from ~32 wt.% in the bulk sample to about ~47 wt.%.

**Figure 3:**
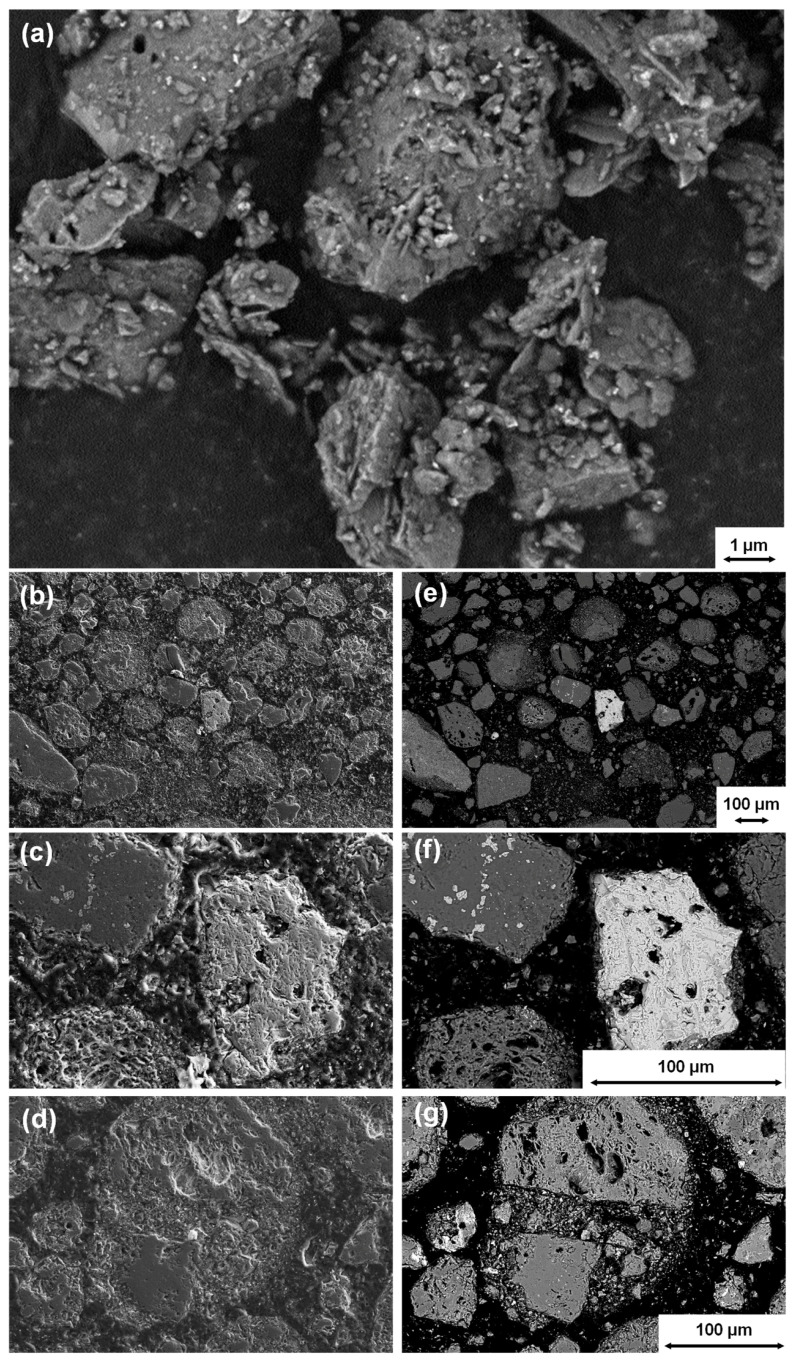
SEM images of ground tailings material with different magnifications ((**a**): SE image of the powder sample, (**b**–**d**): SE images of the polished section of an embedded powdered sample and (**e**–**g**): related BSE images).

**Figure A1:**
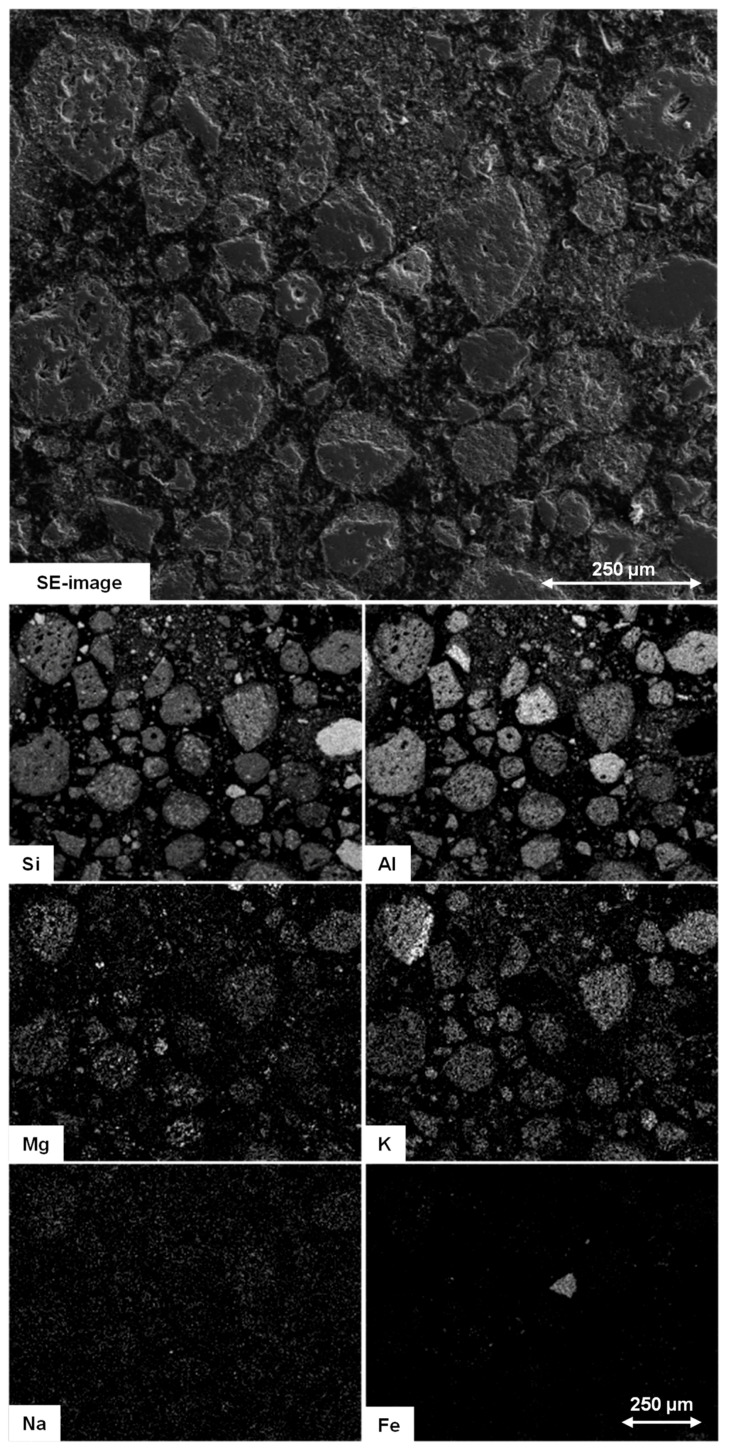
SE image and EDX-mapping for different elements on the polished section of an embedded powdered sample.

Figure 3 contains SEM images of a ground sample. Figure 3a shows layered structures alongside massive grains and reveals that phyllosilicates are still present in their original morphology. Figure 3b–d from a polished section of the embedded powdered sample using a SE detector revealed that most of the particles consist of a porous structure with a broad range of pore sizes from several microns to smaller one micron. This observation appears to be comparable with technical expanded clays [40], in which the water is immediately released from the mineral structure and the intermediate layers resulting in a porous structure. In images from a BSE detector, areas with heavier elements appear brighter. In the BSE images (Figure 3e–g), the bright particles could be identified as hematite by using EDX. In addition to generally very small hematite particles in the range of a few µm, individual larger particles can also be found in the larger grain size range of the powder (~100 µm) (see Figure 3f). Hematite also shows a porous structure, which could be caused by the formation through the release of H_2_O from iron hydroxides or SO_3_ from iron sulfides during heat exposure. In addition, the BSE images show that the individual grains are caked together from different components (e.g., see Figure 3g). This small-range inhomogeneity of the materials is also illustrated in elemental resolution with EDX-mapping for different elements on the polished section of the powdered sample (see Appendix A Figure A1).

The presence of layer silicates and the porous structure of the particles suggest that there could be problems with workability when using the material as SCM. Surprisingly, the workability of mortars with 25 wt.% cement substitution was not significantly affected by most of the samples. In the most negative case, the mortar slump using a Haegermann cone, according to DIN EN 1015-3 [32], was reduced to about 30%, but this was easily adjusted using small amounts of superplasticizer. For samples where the initial flow was not significantly affected, the workability became worse over time compared to the reference, but this effect could also be easily adjusted using small amounts of superplasticizer (PCE basis).

**Figure 4:**
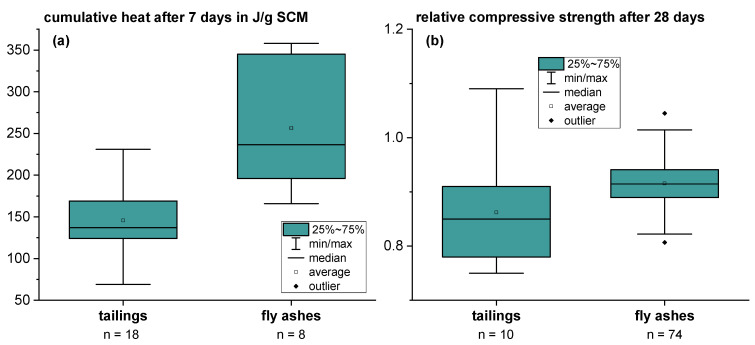
Ranges of 7-day cumulative heat according to ASTM C1897-20 [25] for the tailings materials compared to literature data of siliceous fly ashes [41] (**a**) and 28-day mortar relative compressive strength with a cement substitution level of 25 wt.% for the tailings materials compared to the literature data of siliceous fly ashes [43] (**b**).

Figure 4 shows the ranges of cumulative heat after 7 days according to ASTM C1897-20 [25] (a) and relative compressive strength after 28 days with a cement substitution of 25 wt.% (b) using the tailings materials compared to siliceous fly ashes [41]. The cumulative heat of the investigated tailings materials is mainly in the range of 140–160 J/g SCM and, thus, lower than the literature data for siliceous fly ash [41]. However, individual samples were found to have a reaction heat comparable to siliceous fly ashes. Regarding the relative compressive strength after 28 days, all samples lead to higher values than 0.75 (see Figure 4) and would comparatively fulfill the criteria for fly ashes for use in concrete according to EN 450 [42]. The main data are slightly lower than the data of 74 siliceous fly ashes from reference [43]. However, individual samples of the tailings material lead to high relative compressive strength.

To summarize, the explorative investigations on selective samples from various tailings dumps have shown that the autogenously calcined clays have low to moderate reactivities and are basically suitable as SCM. However, the composition is very inhomogeneous, and the right mining and processing strategy must be selected individually for each tailings dump.

### 3.2. Industrial Trial

#### 3.2.1. Properties of Selected Fractions

**Figure 5:**
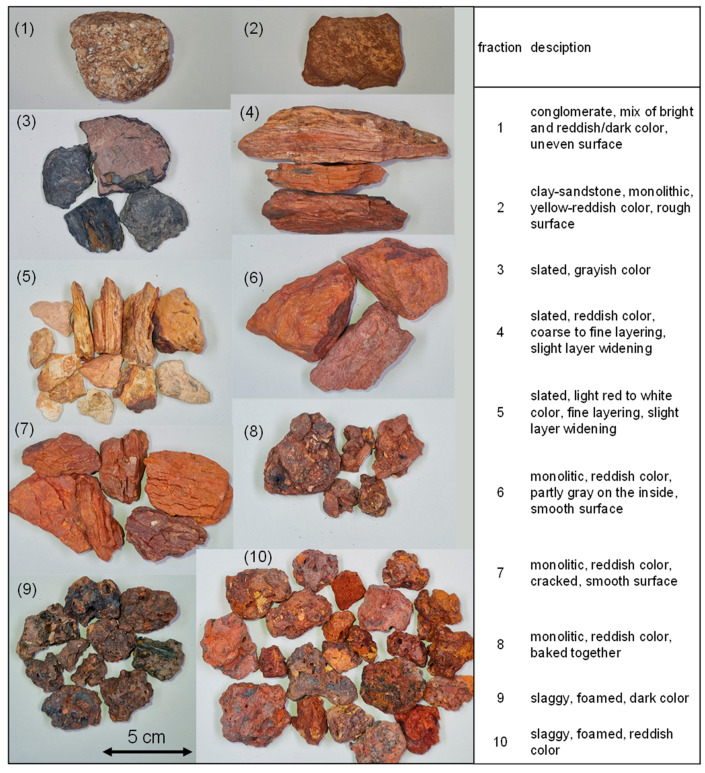
Photos of the selected fractions 1–10 and optical description.

Since the tailings material is very inhomogeneous and constant quality has to be provided in future applications, a closer look was taken at the properties of single fractions contained in the sample for the industrial trial. Figure 5 shows the selected fractions with a short description of the visual characteristics. The bulk sample contains some non-clayey material, such as fraction 1; however, the major part appears to be claystone or shale (fractions 3 to 8). The influence of temperature is visible here in the form of fine widening of the layering or fine pores (fractions 4 and 5), cracks (fraction 7) and even the caking of different grains (fraction 8). Furthermore, some slaggy grains are present, which have high porosity (fractions 9 and 10, porosity ~40 vol.%). These grains are comparable to porous volcanic rock, such as volcanic clinker, and indicate that the material was partially exposed to very high temperatures. The air voids range from <1 mm to >10 mm in the different pieces, indicating variations in the heat exposure (e.g., maximum temperature, exposure time, and heating/cooling rate). Similar observations of temperature altering of the surrounding rock of burning coal seams were made by Grape et al. [3,44]. According to them, fractions 4–8 can be categorized as “clinker” and fractions 9–10 as “paralava”. Moreover, some grains show their original shape on one side, e.g., slate, while on the other side, they are slagged and porous, indicating small-scale, highly heterogeneous temperature conditions. Since most of the grains in the sample show characteristics of more than one of the presented fractions, no mass balance of the different fractions was determined.

The chemical, mineralogical and physical characterization data are listed in Table 9. The mineralogical analysis showed that fraction 1 is mainly quartz and a small proportion of feldspars as common mineral groups of igneous rocks. Small proportions of hematite and the high-temperature mineral mullite and spinel might result from deposition or epitaxial growth through high-temperature metamorphosis of the surrounding rock.

Fraction 2 has the highest layer silicate content and the lowest amorphous content among the other fractions, indicating the lowest temperature exposure compared to fractions 3–10. The latter show varying contents of (recrystallized) high-temperature phases such as mullite, cordierite, spinel and gehlenite. The slaggy fractions (9 and 10) have the highest amorphous content of around 60 wt.%. Interestingly, the fractions 2–10 show quite similar chemical compositions, mainly consisting of SiO_2_, Al_2_O_3_ and Fe_2_O_3_ with a sum average of 89.84 ± 0.87 wt.% (SiO_2_, average = 57.00 ± 4.03 wt.%; Al_2_O_3_, average = 26.87 ± 3.56 wt.%; Fe_2_O_3_, average = 5.97 ± 1.63 wt.%). The only fraction that shows a significant amount of carbon (>0.2 wt.%) is the darkish fraction 3. It is not clear whether the dark color is caused by the carbon or whether it is due to the low hematite content since hematite causes the reddish color of the other fractions [45].

**Figure 6:**
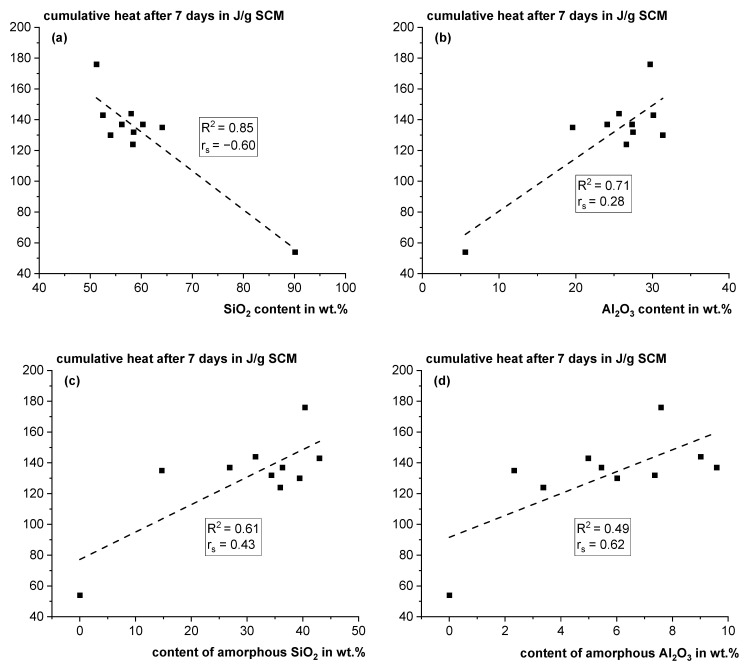
Cumulative heat after 7 days in the reactivity test according to ASTM C1897-20 [25] using isothermal calorimetry as a function of the SiO_2_ (**a**), Al_2_O_3_ (**b**), amorphous SiO_2_ (**c**) and amorphous Al_2_O_3_ (**d**) content (R^2^: coefficient of determination; rs: Spearman correlation coefficient).

Surprisingly, despite the varying characteristics of the tailings material, the reactivity of most of the fractions is quite similar. Fractions 2–8 and 10 have an average cumulative heat release of 135 ± 7 J/g SCM after 7 days in the R^3^ test [25]. Only fraction 9 shows a significantly higher heat release with 176 J/g SCM, which is in the typical range of natural pozzolana or in the lower range of fly ashes [41]. Based on these findings, sorting out quartz-rich unreactive fractions such as fraction 1 would improve the quality of the pozzolan. Multiple regression analyses of the sample characteristics with the R^3^ heat were performed as described in [43,46]; however, no satisfying model could be developed. This could be due to the fact that 80% of the samples (fractions 2–8 and 10) show very similar reaction heat with a relative standard deviation of 4.9 %, and the measurement errors of the different sample characteristics superimpose possible correlations. However, some bilateral relations are discussed in the following. As illustrated in Figure 6a,b, an increasing SiO_2_ content is correlated with decreasing reactivity, whereas an increasing Al_2_O_3_ content is correlated with increasing reactivity. However, this correlation should not be overinterpreted since most of the data are scattered around one point. But, with higher SiO_2_ contents, the probability is increasing that there is a high quartz content and the clay content of the original material was low, and with increasing alumina contents, the probability is increasing that the origin material contained clay phases that can be activated at comparatively low temperatures. However, as discussed in [7] also other frequent phases such as feldspars contain alumina. It is assumed that the X-ray amorphous phase is potentially reactive, as it is a metastable phase due to the low-order state of the atoms, as explained in Section 1. Elements such as Si and Al can be leached in a cementitious environment more easily from this phase than from a stable crystal lattice and are available for the formation of strength-building phases such as C-A-S-H. Figure 6c,d show that the amorphous shares of SiO_2_ and Al_2_O_3_ as a function of the R³ reactivity surprisingly have a higher scatter of the data compared to the bulk contents. However, the X-ray amorphous phases can differ significantly in their near-range order and stability and, thus, also in their reactivity [7]. These differences cannot be detected with XRD, and other methods, such as NMR, are required for a deeper understanding of the structure of X-ray amorphous phases. 

**Figure 7:**
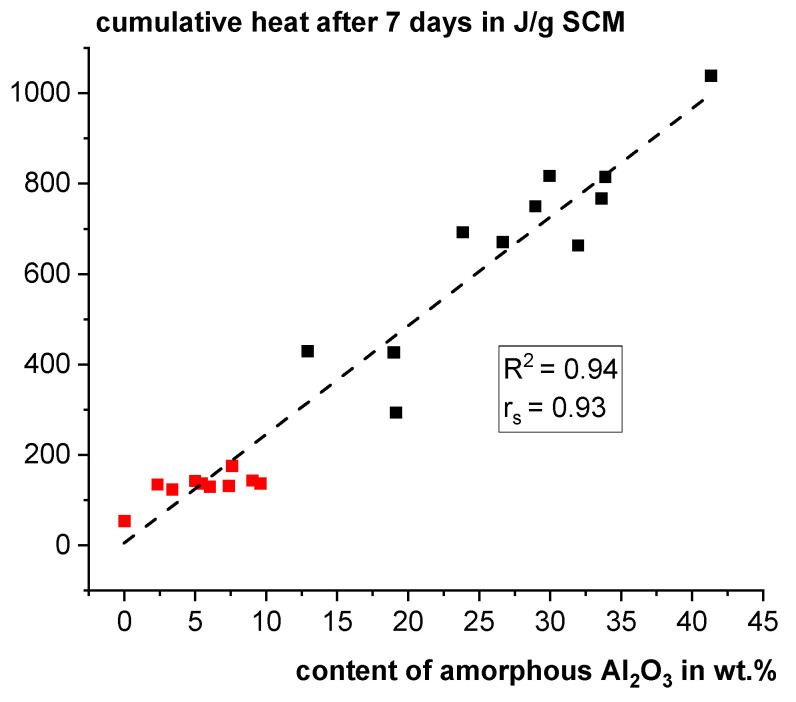
Cumulative heat after 7 days in the reactivity test according to ASTM C1897-20 [25] using isothermal calorimetry as a function of the contents of amorphous Al_2_O_3_ for the ten fractions of Q (red dots) and additional data of calcined clays from [46] (black dots) (R^2^: coefficient of determination; rs: Spearman correlation coefficient).

In contrast, in [46], the content of amorphous Al_2_O_3_ was found to be the decisive factor for the reactivity of calcined clay. In that study, it was assumed that the higher the amorphous Al_2_O_3_ content, the higher the content of meta-kaolinite as the most reactive meta-clay phase. However, the calcined clays in that study had significantly higher amorphous Al_2_O_3_ contents ranging from ~12–42 wt.% with significantly higher reactivity ranging from ~290–1040 J/g SCM. When plotting the data of the ten fractions of Q together with the data from [46], it is revealed that the strong scatter is just a scale effect (see Figure 7). There is a strong overall correlation with a Spearman correlation coefficient of 0.93 and a coefficient of determination for linear regression of 0.94. It can be assumed that in the small range with comparatively low reactivity of the ten fractions of Q, the influence of non-measurable physical properties such as grain morphology and structure (see SEM investigations in Section 3.1) could have a decisive impact on reactivity leading to the bad correlations with other measured characteristics.

**Figure 8:**
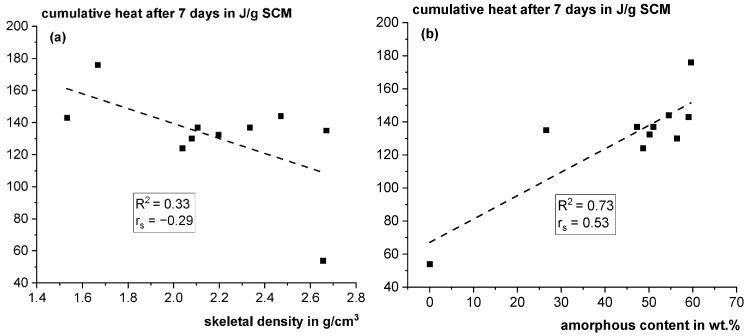
Cumulative heat after 7 days in the reactivity test according to ASTM C1897-20 [25] using isothermal calorimetry vs. skeletal density (**a**) and amorphous content (**b**) (R^2^: coefficient of determination; rs: Spearman correlation coefficient).

Another interesting observation was the strong difference in the skeletal density of the different fractions of Q. Figure 8a shows that the skeletal density only weakly correlates with reactivity. The amorphous content also shows a correlation to reactivity; however, due to the scatter, it cannot be used as a good indicator to predict reactivity (see Figure 8b).

**Figure 9:**
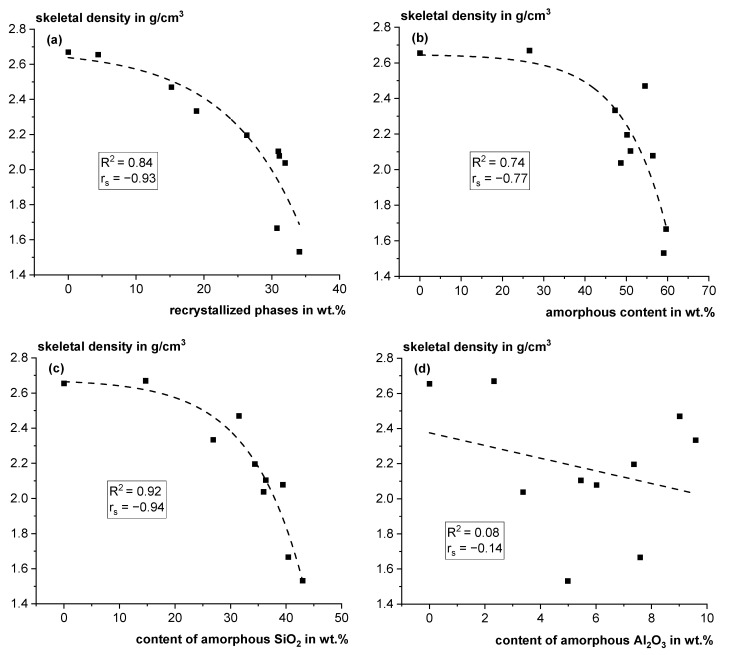
Skeletal density as a function of the content of recrystallized phases (**a**), the amorphous phase content (**b**), the amorphous SiO_2_ content (**c**) and the amorphous Al_2_O_3_ content (**d**) (R^2^: coefficient of determination; rs: Spearman correlation coefficient).

Figure 9a,b show that the skeletal density correlates with the sum of recrystallized phases and the amorphous content using exponential functions. The correlations have high Spearman correlation coefficients of −0.93 and −0.77, respectively. Although the skeletal density is not a good parameter to predict reactivity, a low density clearly indicates that the material was exposed to elevated temperatures and suggests that it originally contained clay phases that released H_2_O, inducing the porous structure. Interestingly, the skeletal density can also be correlated with the amorphous SiO_2_ content (see Figure 9c), with a Spearman correlation coefficient of −0.94, but not with the amorphous Al_2_O_3_ content (see Figure 9d). Accordingly, also the total amorphous content can be correlated with the amorphous SiO_2_, while the amorphous Al_2_O_3_ content is not correlated. This results from the recrystallization of Al-rich phases like mullite, cordierite and spinel. The amorphous SiO_2_/Al_2_O_3_ ratios of the ten fractions of Q differ in the range of ~3–11, whereas for the calcined clays in [46], they are significantly lower in the range of ~1–2. If meta-clay phases with different reactivities are assumed in the amorphous share of the sample, as discussed in [7], the recrystallization of phases with high alumina content would lead to a change in the metaphases and alter the reactivity of the residual structure. The effects of recrystallization on the reactivity of the remaining amorphous phase need to be further investigated.

#### 3.2.2. Industrially Produced Cements

##### Isothermal Calorimetry

**Figure 10:**
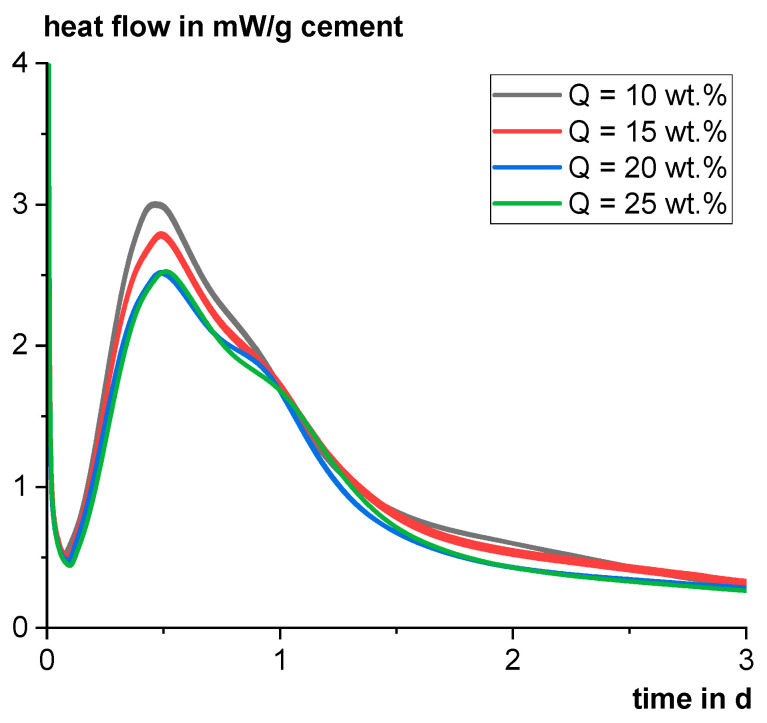
Heat flow of the industrially produced cements with different percentages of the tailings material (Q).

The heat flow of the different industrially produced cements in the first three days with a w/c ratio of 0.5 is illustrated in Figure 10. The higher the content of Q, the lower the maximum of the C_3_S hydration peak and the lower the cumulative heat. However, CEM II/B-Q20 and CEM II/B-Q25 behave quite similarly. The beginning of hydration is also slightly delayed with rising Q content. Basically, the effect of Q on the heat flow curves is weak, indicating only low reactivity within the first three days. However, the results also indicate that Q has no significant negative influence on clinker hydration. To substantiate this statement, the effect on hydration is studied in more detail in the following chapter.

##### Hydration Study

**Figure 11:**
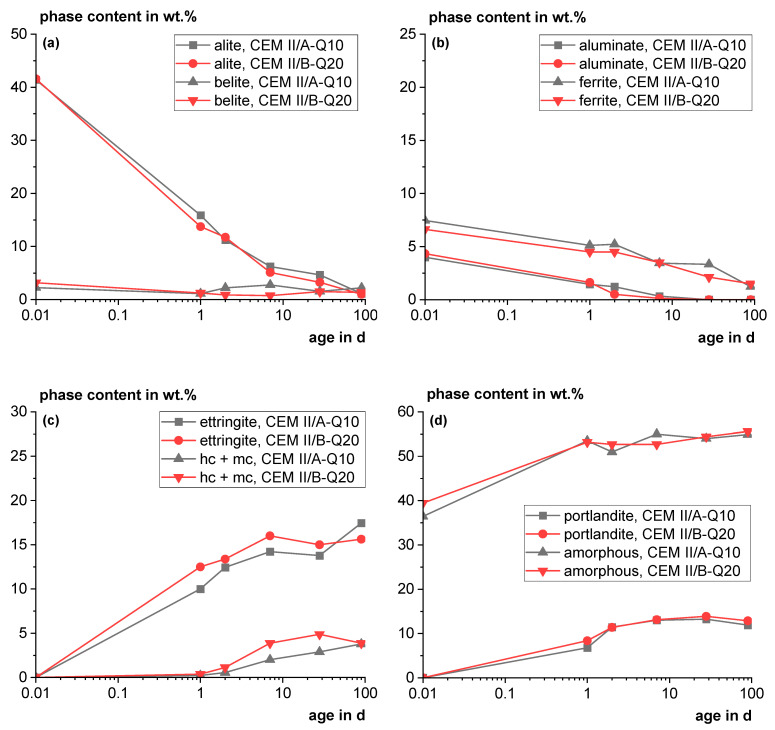
Phase contents as a function of time; (**a**,**b**): decrease in the clinker phases, (**c**,**d**): formation of the main hydration products for the industrial trial cements CEM II/A-Q10 and CEM II/B-Q20 normalized to the cement without Q (except for the amorphous content).

Figure 11 shows the phase development as a function of time of the main phases for the industrial trial cements CEM II/A-Q10 and CEM II/B-Q20. For illustration and comparability, the phase contents of the cements were normalized to 100 wt.% cement without Q (except the amorphous content).

The alite reaction shows no distinct difference between the cements (see Figure 11a). The Q content does not appear to influence the alite dissolution significantly. Within the accuracy of the method, the belite content of the industrial trial cements, which was very low from the beginning, seems to be more or less constant during the whole observed 90 days (see Figure 11a). The slow belite reaction is also known from the literature, e.g., in [47,48]. The aluminate (C_3_A) content decreases quickly and cannot be detected after 7 days (see Figure 11b). Ferrite (brownmillerite) decreases slowly and can still be detected after 90 days (see Figure 11b). The ettringite content seems to rise slightly in the overall trend (see Figure 11c). The CEM II/B-Q20 shows a slightly higher formation of carboaluminate hydrate phases compared to the CEM II/B-Q10, which might indicate the slight reactivity of the alumina of the tailings material and the synergetic effect by the reaction with the calcium carbonate contained [49] (see Figure 11c). The portlandite content seems to decrease slightly from 28 to 90 days, indicating a slight pozzolanic reaction (see Figure 11d).

In summary, the results of the hydration study indicate a slight reactivity; however, the error of the method has to be considered. On the other side, no negative effect on hydration by using the tailings material was observed either.

##### Setting Time, Soundness and Mortar Experiments

**Figure 12:**
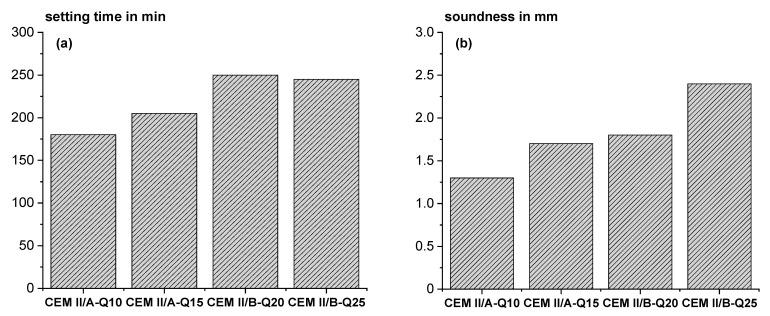
Setting time (**a**) and soundness (**b**).

Figure 12 shows the setting time and soundness according to EN 196-3 [31]. A slight delay in setting time occurs with increasing Q content (see Figure 12a), which is consistent with the previous calorimetry results. However, it is comparable to other standardized SCMs. The soundness tends to increase slightly with increasing Q content (Figure 12b) but is far below the threshold value of 10 mm according to EN 197-1 [50].

**Figure 13:**
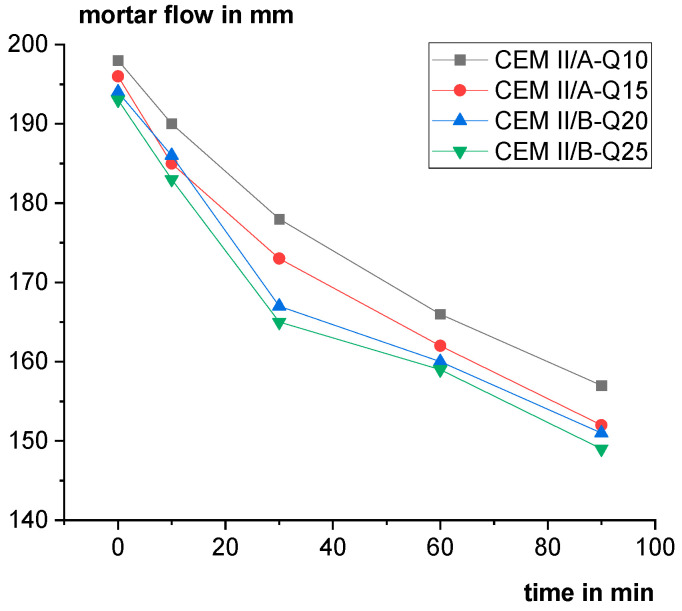
Mortar flow as a function of time.

Figure 13 shows the mortar flow as a function of time for the different cements. Over time, the mortar flow is decreasing. The higher the Q content is, the lower the mortar flow. However, with small additions of superplasticizer the mortar flow could be adjusted easily.

**Figure 14:**
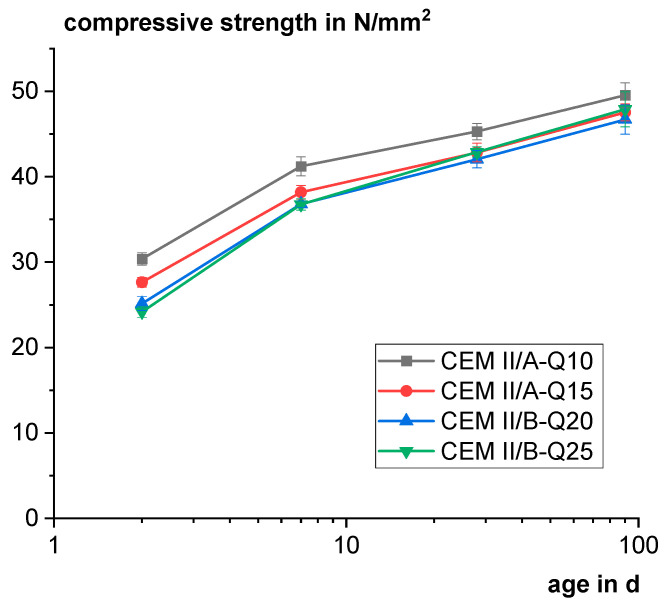
Mortar compressive strength of the industrially produced cements with different content of Q.

Figure 14 shows that with a higher content of Q, the compressive strength decreases. The effect is most pronounced at an early age of 2 days. The differences become smaller with increasing age, showing the slight pozzolanic reactivity of Q. Especially from 28 days on, there is no significant difference between 15 and 25 wt.% Q considering the standard deviation of the measurements.

The results show that the material is generally suitable for use as a cement component, but the processing costs, scalability and quality control effort must be evaluated for each specific project. Generally, the use of the tailings faces comparable challenges as for other natural pozzolans with regard to mining permits, possible processing steps, transportation routes and grinding. The business case must weigh up the costs of different levels of processing against the market requirements. Furthermore, environmental compatibility has to be ensured in the frame of quality control. Since the tailings are basically natural clayey sediments, no substantial leaching of environmentally relevant substances is to be expected (compare Appendix A Table A1). However, caution is required in the case of landfills in which other waste, such as coking plant waste, has also been deposited.

#### 3.2.3. Additional Investigations on Model Mixtures

##### Reactivity Test (R^3^) Using Isothermal Calorimetry

**Figure 15:**
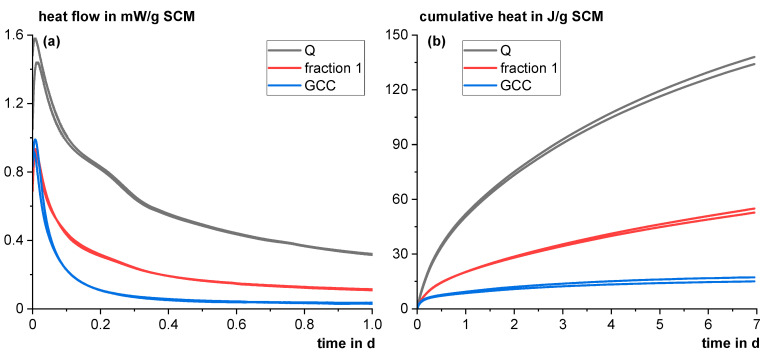
Heat flow in the first day of reaction (**a**) and cumulative heat up to 7 days (**b**) in the R^3^ test according to ASTM C1897-20 [25] for Q compared to considered inert materials (faction 1: mainly quartz, see Section 3.2.1; GCC: ground calcium carbonate).

In the first step, the reactivity of Q ground on a laboratory scale for the model mixtures was tested using the R^3^ test according to ASTM C1897-20 [25]. It showed an average cumulative heat of 136 J/g SCM which is in good agreement with the average of the ten investigated fractions of Q in Section 3.2.1. However, it is in the lower reactivity range of the tailings materials already investigated in Section 3.1 (see Figure 4). Compared to the literature data [41], it is also in the lower reactivity range of natural pozzolans. However, compared to mostly inert materials such as ground calcium carbonate (GCC) or fraction 1 from Section 3.2.1 (mainly quartz), it clearly shows a higher reactivity in the R^3^ test (see Figure 15).

##### Hydration Study

**Figure A2:**
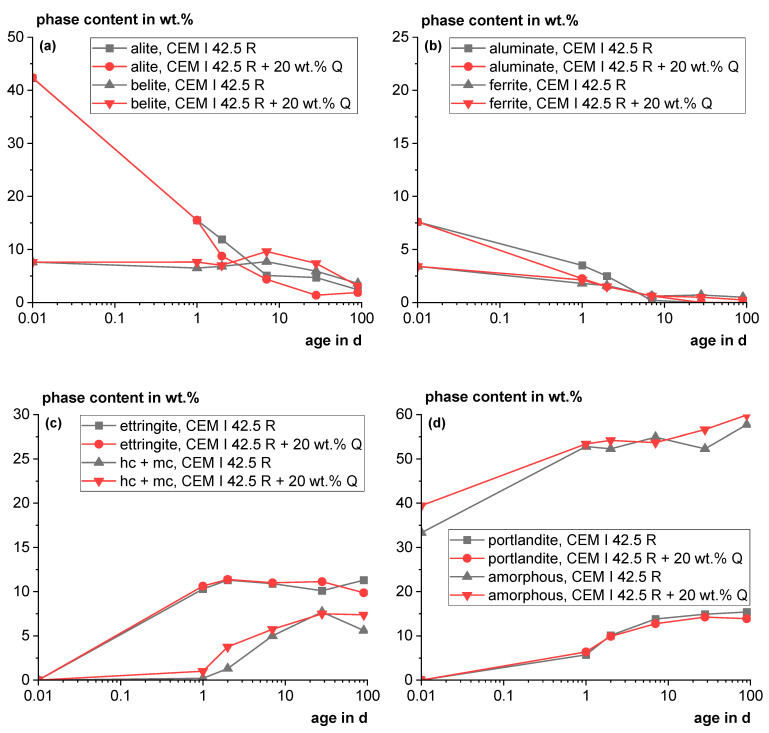
Phase contents as a function of time of the clinker phases (**a**,**b**) and the main hydration products (**c**,**d**) for the model mixture containing 20 wt.% Q normalized to 100 % cement (except the amorphous content) and the reference cement. (For illustration, in the logarithmic scale, the time point zero was set to 0.01 days.).

The hydration study was also performed on the model mixtures using the commercial CEM I 42.5 R substituted with 20 wt.% by the pozzolan Q compared to the reference with 100 wt.% CEM I 42.5 R similar to the hydration study of the industrially produced cements in Section 3.2.2. This was done in order to have a comparison with a reference without Q content. The results can be found in the Appendix A Figure A2. The phase developments are quite similar to those of the industrial cements. Without considering inaccuracies in the results, a portlandite consumption of 2.6 g/100 g Q after 28 days and 6.1 g/100 g Q after 90 days were determined, showing a slight pozzolanic reactivity.

##### Mortar Compressive Strength

**Figure 16:**
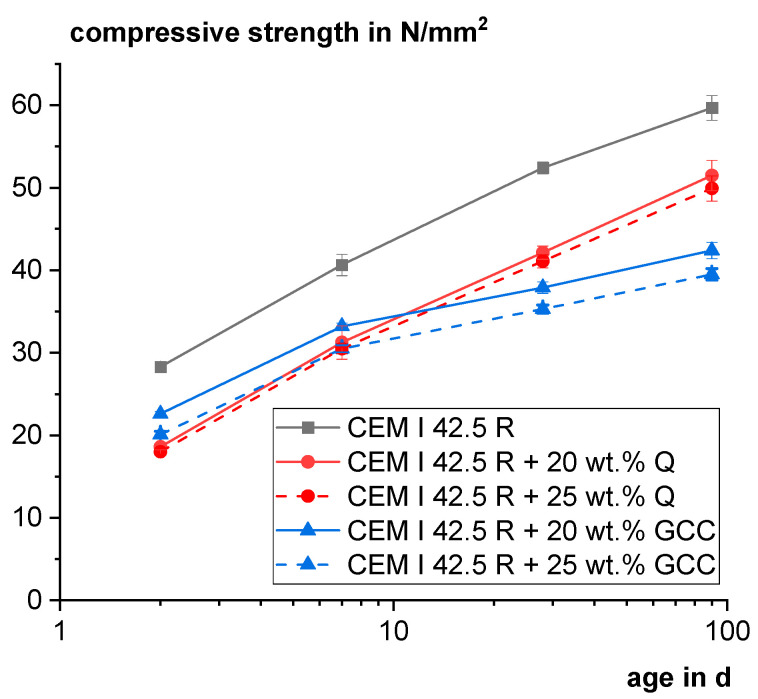
Comparison of mortar compressive strength of binary mixtures with the pozzolan Q and ground calcium carbonate (GCC).

Figure 16 shows the mortar compressive strength of model mixtures to compare the performance of Q with typical quasi-inert GCC. Because of its very high fineness, GCC leads to a higher early age strength than Q. After 28 and 90 days, the blends with Q clearly outperform the GCC blends, demonstrating the pozzolanic reactivity. The 25 wt.% Q blend reached relative compressive strengths of 0.78 and 0.84 after 28 and 90 days, respectively.

**Figure 17:**
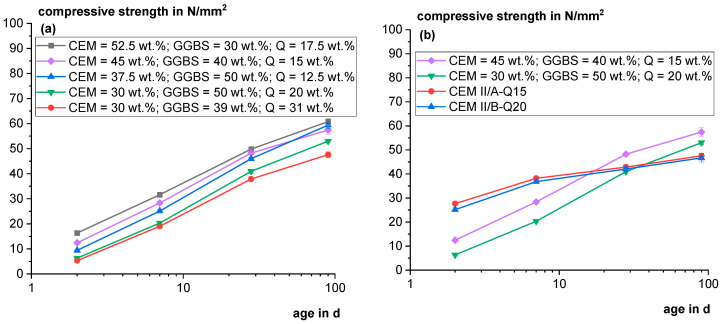
Development of compressive strength of ternary model mixtures (CEM: ~88 wt.% clinker, ~6 wt.% sulfates and ~6 wt.% limestone) (**a**) and a comparison of binders with 15 and 20 wt.% Q (**b**).

For the outlook, ternary systems with a further reduced clinker factor were also examined. Figure 17a shows the compressive strength of different mixes of ternary systems. The composition of the binder fraction is indicated in the figure. The lower the clinker content, the lower the overall strength, which is also true when increasing the GGBS content and lowering the Q content. When comparing both mixes with the lowest clinker content, it becomes clear that substituting GGBS with Q leads to lower strength. However, also considering that the sulfate content was not adjusted properly in the experiments and the grain size distributions of the components were not optimized, it becomes obvious that cements in the strength class 32.5 N can be produced with ultralow clinker factor (CEM V/B) using high proportions of the comparatively low reactive tailings sample. Figure 17b shows the mortar compressive strength of the industrially produced cements compared to the mixes using GGBS containing the same content of Q. It becomes visible that the initial strength in the mortars with GGBS is strongly reduced, but the late strength is higher.

## 4. Conclusions

Concerning their availability, the burned tailings dumps represent a huge resource of material for the construction industry, not only in Germany but worldwide in coal mining regions. However, the composition of the material is complex and depends on the origin and the burning conditions and will, therefore, differ in different dumps and especially in different regions.

In this study, tailings materials from different locations in western Germany were investigated, showing that the material is basically suitable as an SCM, as it consists mainly of SiO_2_ and Al_2_O_3_, which partially occurs in X-ray amorphous form and has a low content of impurities such as carbon or sulfur. The R^3^ reactivity and the relative mortar compressive strength in a cement system differed significantly depending on the sample.

An industrial cement production trial was conducted with low processed tailings material. It showed good grindability and processability in cement manufacturing; however, due to the relatively low reactivity, it showed a comparatively low strength contribution.

The overall results suggest that with an adjustment of particle size distributions and formulations, practically suitable cements and concrete can also be produced when using low-reactive autogenously calcined clays from coal tailings. Further research is required into the variability of its properties and the factors determining reactivity to develop effective processing strategies that can be adapted from one dump to another. Also, the impact of the tailings material on the durability of concrete needs to be investigated.

## Figures and Tables

**Table 1:** Processes during calcination summarized from the literature [7].

Process	Kaolinite	Montmorillonite	Illite
	Temperature in °C		
dehydroxilation	~450–700	~600–800	~450–700
amorphization		~800–900	~900
beginning sintering/melting	-	~900	
beginning recrystallization	~925		~1000

**Table 2:** Labeling of the samples with description and assignment to the corresponding chapters.

Sample	Description	Section
samples from exploratory investigations	not specified individually because of confidentiality	Section 3.1
fraction 1–10	handpicked fractions of Q	Section 3.2.1
Q	tailings sample for the industrial cement production trial	Section 3.2.2
CEM II/A-Q10	CEM II/A-Q with 10 wt.% from Q from the industrial cement production trial	Section 3.2.2
CEM II/A-Q15	CEM II/A-Q with 15 wt.% from Q from the industrial cement production trial	Section 3.2.2
CEM II/B-Q20	CEM II/B-Q with 20 wt.% from Q of the industrial cement production trial	Section 3.2.2
CEM II/B-Q25	CEM II/B-Q with 25 wt.% from Q of the industrial cement production trial	Section 3.2.2
CEM I 42.5 R	test cement for model mixtures	Section 3.2.2
GCC	ground calcium carbonate for model mixtures and reactivity testing	Section 3.2.2
GGBS	ground granulated blast furnace slag for model mixtures	Section 3.2.2

**Table 3:** Mineralogical composition of the raw materials and cements from the industrial trial.

Phase	Unit	Q	Sulfate Carrier	Limestone	Clinker	CEM II/A-Q		CEM II/B-Q	
						Q = 10%	Q = 15%	Q = 20%	Q = 25%
alite	wt.%	-	-	-	71.3	55.9	49.7	49.9	45.8
belite					8.0	3.1	4.2	3.8	3.3
aluminate (C_3_A)					6.3	5.4	4.6	5.2	4.2
brownmillerite					13.2	10.1	8.1	7.9	7.6
arcanite					1.3	1.3	1.3	1.5	1.0
anhydrite			54.5		-	6.4	7.2	5.4	6.2
bassanite			-			-			
gypsum		1.4	24.8			1.4	1.4	1.1	1.5
calcite		-	0.6	89.6		4.2	5.9	3.1	4.2
dolomite			8.1	-		0.4	0.4	0.4	0.4
quartz		22.6	5.0	4.4		3.6	5.5	6.3	7.0
illite/muskovite		9.3	3.2	6.1		1.4	1.9	2.2	2.6
hämatite		1.6	-	-		0.2	0.2	0.3	0.4
feldspar		3.3	3.8			0.6	0.7	0.9	1.0
mullite		11.6				1.1	1.6	2.1	2.6
codierite		2.2				0.2	0.3	0.4	0.4
spinel		1.4				0.1	0.2	0.3	0.3
corundum		0.6				0.1	0.1	0.1	0.2
amorphous		46.0				4.6	6.9	9.2	11.5

**Table 4:** Mineralogical composition of the additional raw materials for the model mixtures.

Phase	Unit	CEM I 42.5 R	GCC	GGBS
alite	wt.%	63.5	-	-
belite		11.4		
aluminate (C_3_A)		11.4		
brownmillerite		5.1		
arcanite		-		
anhydrite		2.1		
bassanite		1.6		
gypsum		2.4		
calcite		1.7	95.8	
dolomite		-	3.7	
quartz		0.7	0.5	
amorphous		-	-	100 ^1^

^1^ no reflexes.

**Table 9:** Characterization data of the ten fractions.

Parameter	Unit	Fraction									
		1	2	3	4	5	6	7	8	9	10
loss on ignition	wt.%	0.32	2.60	3.98	0.89	0.81	0.83	0.60	0.80	0.37	0.64
total sulfur as SO_3_		0.08	0.04	0.63	0.21	0.30	0.35	0.18	0.17	0.28	0.38
total carbon as C		0.05	0.03	2.73	0.03	0.03	0.06	0.06	0.05	0.17	0.09
SiO_2_ + Al_2_O_3_ + Fe_2_O_3_		99.81	89.99	87.84	90.68	89.57	90.94	89.80	89.73	89.92	90.11
Na_2_O		0.02	0.10	0.01	0.01	0.06	0.08	0.11	0.03	0.20	0.07
K_2_O		0.70	3.55	4.81	4.73	6.24	3.91	5.09	4.91	5.25	4.92
MgO		0.22	1.49	1.48	1.46	1.02	1.91	1.80	1.63	2.52	2.18
Al_2_O_3_		5.60	19.58	25.64	27.45	31.34	24.10	27.33	26.59	29.69	30.10
SiO_2_		90.11	64.09	57.99	58.47	53.96	60.29	56.16	58.33	51.21	52.46
P_2_O_5_		0.06	0.10	0.06	0.06	0.06	0.05	0.05	0.05	0.03	0.04
CaO		0.20	0.31	0.21	0.21	0.49	0.18	0.31	0.21	0.71	0.70
TiO_2_		0.25	0.98	1.13	1.10	1.17	1.14	1.13	1.09	1.09	1.12
MnO		0.06	0.16	0.09	0.09	0.05	0.10	0.11	0.08	0.21	0.16
Fe_2_O_3_		4.10	6.32	4.21	4.76	4.27	6.55	6.30	4.81	9.03	7.54
illite/muskovite	wt.%	n.d.	39.7	12.2	4.1	3.6	4.4	n.d.	1.2	3.4	2.6
quartz		90	31.4	16.5	13.2	4.5	27.9	8.7	15.4	n.d.	n.d.
feldspar		2.8	1.3	1.5	4.1	2.5	n.d.	5.6	1.0	3.0	2.2
gypsum		n.d.	n.d.	n.d.	n.d.	n.d.	n.d.	0.7	n.d.	n.d.	n.d.
hematite		2.7	1.1	0.2	2.3	2.1	1.6	3.1	1.8	3.3	2.2
mullite		3.2	n.d.	8.1	16.5	26.1	11.2	18.1	21.9	11.2	17.1
cordierite		n.d.	n.d.	1.3	5.9	1.4	1.5	7.2	2.0	8.8	5.4
gehlenite		n.d.	n.d.	n.d.	1.0	n.d.	2.4	0.2	0.5	0.7	0.5
spinel		1.2	n.d.	5.3	1.8	1.5	3.8	4.1	7.6	8.5	10.1
corundum		n.d.	n.d.	0.6	1.3	2.2	n.d.	1.5	n.d.	1.7	1.1
amorphous		n.d.	26.6	54.5	50.2	56.4	47.3	51.0	48.7	59.6	59.1
Blaine	cm^2^/g	4469	6954	7898	4897	4203	3353	5423	3547	4812	3549
BET surface area		12,800	71,900	47,700	24,700	19,900	25,700	18,400	15,700	15,300	14,900
D_10_	µm	1.1	1.2	1.0	0.8	0.7	0.7	0.9	2.1	0.7	1.5
D_50_		11.2	9.3	8.3	6.9	7.2	5.9	9.7	28.1	6.2	23.8
D_90_		72.6	52.1	48.5	50.2	49.7	37.8	69.6	97.2	40.1	94.2
true density	g/cm^3^	2.70	2.76	2.64	2.66	2.65	2.71	2.68	2.62	2.70	2.68
skeletal density		2.66	2.67	2.47	2.20	2.08	2.33	2.10	2.04	1.67	1.53
porosity	-	1.6	3.3	6.4	17.4	21.6	13.9	21.5	22.2	38.3	42.8
cumulative heat after 7 d [25]	J/g SCM	54	135	144	132	130	137	137	124	176	143

n.d.: not detected.

## Data Availability

Data are contained within this article.

## Appendix A

**Table A1:** Range of eluate parameters according to EN 12457-4 of the tailings compared to German threshold values for the landfilling of non-hazardous wastes [51].

Parameter	Unit	Range (Tailings)	Thresholds Landfill Class 0 [51]
pH value	-	7.68–10.94	5.5–13
el. conductivity	µS/cm	162–614	-
chloride	mg/L	<0.1–0.3	80
sulfate		36–191	100
Sb	µg/L	<1–1	6
As		13–67	50
Ba		17.2–27.6	2000
Pb		<1	50
B		30–50	-
Cd		<0.2	4
Cr		<1–10	50
Co		<0.2–0.5	-
Cu		1–11	200
Mo		5–6	50
Ni		<1–4	40
Hg		<0.1	1
Se		1–4	10
Tl		<0.2	-
V		7–19	
Zn		5–10	400
